# The Short Term Influence of Chest Physiotherapy on Lung Function Parameters in Children With Cystic Fibrosis and Primary Ciliary Dyskinesia

**DOI:** 10.3389/fped.2022.858410

**Published:** 2022-05-23

**Authors:** Bjarne Vandervoort, Django De Beuckeleer, Elke Huenaerts, Marianne Schulte, François Vermeulen, Marijke Proesmans, Thierry Troosters, Myriam Vreys, Mieke Boon

**Affiliations:** ^1^Department of Rehabilitation Sciences, Catholic University of Leuven, Leuven, Belgium; ^2^Department of Pediatrics, University Hospital Leuven, Leuven, Belgium; ^3^Department of Development and Regeneration, Catholic University of Leuven, Leuven, Belgium

**Keywords:** airway clearance, physiotherapy, bronchiectasis, primary ciliary dyskinesia, cystic fibrosis

## Abstract

Airway clearance therapy (ACT) is one of the cornerstone treatment modalities to improve mucociliary clearance for patients with bronchiectasis. The progression of lung disease in patients with bronchiectasis can be evaluated by spirometry and multiple breath washout (MBW) and it is advised to monitor these on a regular basis. However, the short term effect of ACT on spirometry and MBW parameters is insufficiently clear and this variability may impact standardization. For cystic fibrosis (CF), available literature refutes a short time effect on spirometry and MBW parameters in children, however, for primary ciliary dyskinesia (PCD) no data are available. We performed a single-center, prospective cross-over study to evaluate the short term effect of a single ACT session using positive expiratory pressure mask on forced expiratory volume in 1 s (FEV_1_) and lung clearance index (LCI), derived from MBW, compared to no ACT (control) in pediatric patients with CF and PCD. A total of 31 children were included: 14 with PCD and 17 with CF. For the whole group, there was no difference in median change of FEV_1_ pp between the treatment and the control group (*p* 0.969), nor in median change of LCI (*p* 0.294). For the CF subgroup, the mean change in FEV_1_ pp with ACT was −1.4% (range −9 to + 5) versus −0.2% (range −6 to + 5) for no ACT (*p* 0.271), the mean change in LCI with ACT was + 0.10 (range −0.7 to + 1.2) versus + 0.17 (range −0.5 to + 2.8) for no ACT (p 0.814). In the PCD subgroup, the mean change in FEV_1_ pp with ACT was + 1.0 (range −7 to + 8) versus −0.3 (range −6 to + 5) for no ACT (*p* 0.293) and the mean change in LCI with ACT was −0.46 (range −3.7 to + 0.9) versus −0.11 (range −1.4 to + 1.3) for no ACT (*p* 0.178). There was no difference between PCD and CF for change in FEV_1_ pp after ACT (*p* = 0.208), nor for LCI (*p* = 0.095). In this small group of pediatric patients, no significant short-term effect of chest physiotherapy on FEV_1_ pp nor LCI in PCD and CF values nor variability was documented.

## Introduction

Mucociliary clearance (MCC) is one of the most important defense mechanisms of the human airway against infections or inhaled pollution (1). Cilia lining the respiratory epithelium are an essential part of the MCC and move the mucus layer toward the pharyngeal cavity. Disorders of the MCC such as primary ciliary dyskinesia (PCD) and cystic fibrosis (CF) result in chronic airway inflammation, infection and eventually airway damage (2). This results in the formation of bronchiectasis with chronic cough and sputum production (1).

Cystic fibrosis (CF) is caused by biallelic mutations in the Cystic Fibrosis Transmembrane conductance Regulator (*CFTR*) gene (3), which translates to the CFTR protein, a transmembrane chloride channel mainly present in the respiratory and gastro-intestinal epithelium. Impaired chloride and water transport disturb the airway MCC by altering the periciliary liquid layer and the viscosity of the mucus layer. The defective MCC causes chronic airway infection and obstructive lung disease worsening with age, leading to respiratory insufficiency in young adults.

Primary ciliary dyskinesia (PCD) is a more rare inherited disease caused by abnormalities in the function and/or structure of the motile cilia, especially in the upper and lower respiratory tract. Patients suffer from chronic upper and lower respiratory tract infections, and almost half of them have situs inversus (4). While CF is characterized by a “chemical” disturbance of the MCC, in PCD the MCC disturbance has a “mechanical” origin (1).

The respiratory treatment for both disorders mainly consists of enhancement of the mucociliary clearance by physiotherapy and inhaled mucolytics combined with antibiotic treatment for acute and chronic infections (5, 6). Current physiotherapy management of CF is multifaceted, inclusive of a combination of inhalation therapy, airway clearance therapy (ACT), physical education/exercise and ongoing education about the disease and its treatment. ACT has the goal to facilitate mucus expectoration from the airways, by applying manual compression techniques, autogenic drainage and stimulating cough and huff (open glottis): by increasing the expiratory flow, secretions are more easily moved toward the oropharynx.

Positive expiratory pressure devices are used to prevent airway collapse and to recruit non-ventilated areas with occluded airways and to facilitate mucociliary clearance.

As progressive worsening of obstructive lung disease is frequent in patients with bronchiectasis, strict monitoring of disease progression with regular lung function assessment is advocated as good clinical practice (2).

Spirometry is the most frequently used, and a well standardized technique to monitor airway disease. Progressive decline in forced expiratory volume in 1 s (FEV_1_) is observed in both disorders (7) and related to morbidity and mortality. In recent years, measures of ventilation heterogeneity such as lung clearance index (LCI) measured by the multiple breath washout test (MBW), have gained more attention. LCI seems to be more sensitive than spirometry for early airway disease (8, 9) and correlates with the risk of respiratory exacerbations (10, 11).

Standardization of lung function measurements is of utmost importance for correct assessment of a treatment effect, both for long-term follow-up of lung disease, and for use in clinical trials. Guidelines for standardization of spirometry and MBW report on required quality criteria for acceptability, repeatability and variability (12, 13). However, the timing of measurements in relation to ACT is not mentioned and not well studied. Previous studies in patients with CF showed heterogeneous results, with a significant effect on FEV_1_ mainly in adults with advanced disease, but not on LCI (14–17). In PCD, there is only one small study in children that showed a heterogeneous change in FEV_1_ after exercise (18).

The reported studies were characterized by different designs, different ACT techniques and their combination with for instance a bronchodilator or a treadmill. In addition, most studies lacked a control group.

As stimulation of mucus clearance may change the airflow obstruction and ventilation inhomogeneity, assessment of the effect of ACT on FEV_1_ and LCI is clinically relevant. A proper control arm is important, as sputum expectoration is stimulated by the lung function maneuvers itself.

The aim of the present study was to study the short-term influence of ACT, more specifically using a positive expiratory pressure (PEP) mask, on spirometry (FEV_1_) and measures of ventilation heterogeneity (LCI by MBW) in children with CF and PCD, in a cross-over study.

We hypothesize a significant short-term effect of ACT on lung function measurements in PCD, as the disturbance in MCC is of a mechanical character. In CF, where the defect is more of a chemical nature, we expect less impact of ACT to induce changes in lung function parameters.

## Materials and Methods

### Subjects

Children between 6 and 18 years of age with a confirmed diagnosis of CF or PCD and able to perform spirometry were recruited in the Pediatric Pulmonology Department of the University Hospital of Leuven for a prospective cross-over clinical trial between June 2020 and June 2021. The diagnosis of CF was confirmed by a sweat chloride value ≥ 60 mmol/L and/or the presence of two disease causing mutations in the *CFTR* gene. All patients were pancreatic insufficient and had lung disease. The diagnosis of PCD was confirmed according to the European guidelines (19). A nasal punch biopsy, including a cell culture to exclude secondary defects was performed in all patients for functional evaluation of ciliary motility with high speed videomicroscopy and ultrastructural evaluation with transmission electron microscopy (20). Genetic analysis was performed in most patients.

Patients had to be respiratory stable at the time of inclusion, defined as “no change in sputum, no fever, no change in therapy for 30 days prior to inclusion, no more than 10% change in FEV_1_ pp since the last visit.”

Height, weight and body mass index (BMI) were expressed as z-scores according to the Flemish reference equations (21).

The study was approved by the research ethics committee UZ/KU Leuven (s55766). Informed consent/assent was obtained from the patients (if > 12 years) and/or their parents.

### Study Design

**FIGURE 1:**
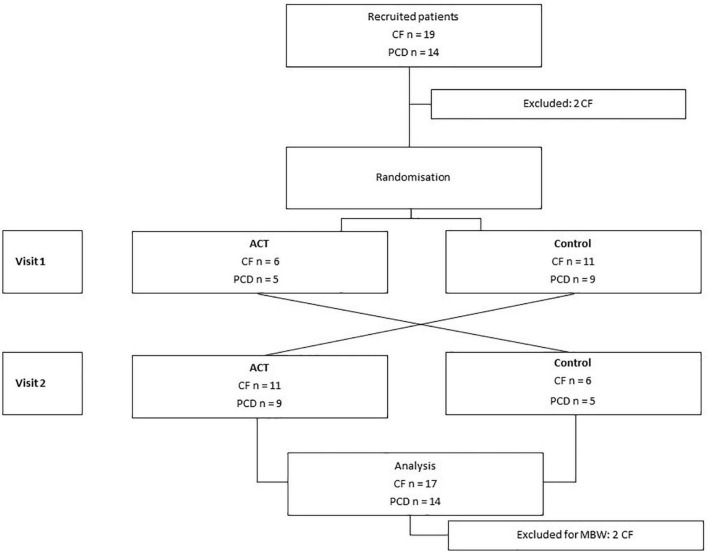
Study overview. A total of 33 patients were recruited to participate in the study: 19 with CF, 14 with PCD. Two patients with CF were excluded. Patients were randomly selected before visit 1 for ACT or the control visit. After the first visit, cross-over to the other study intervention was done. In total, data from 17 patients with CF and 14 with PCD were available for spirometry, 15 with CF and 14 with PCD for MBW.

We performed a single-center, prospective, randomized cross-over study. Patients were recruited at the time of a scheduled routine visit. At the first visit, patients were randomly assigned to the intervention (airway clearance therapy, ACT) or the control group (Figure 1). Due to the nature of the intervention blinding was not possible. Cross-over to the second visit after 6 months (= next scheduled visit) resulted in paired data for each individual patient.

The intervention consisted of a standardized session of ACT: a positive expiratory pressure (PEP) mask was used with the help of a physiotherapist. Three series of PEP were executed, each with 20 expirations followed by a minimum of three huffs and expectoration of mucus. Manual external compression of the chest by the physiotherapist was added to stimulate the process of mucus clearance. Every patient used his/her personal PEP mask with corresponding resistance to ensure comfort and custom-fit material. The resistance used was the one for which the patient could sustain a pressure of 15 cm of water for about 2 min of tidal breathing. No bronchodilators or other inhaled drugs were administered prior or during the ACT. Patients did not perform prior physiotherapy on the day of the study.

For the control condition, patients were allowed a period of 30 min of rest.

### Outcome Measures

The spirometry-derived parameters FVC and FEV_1_ were assessed using a hand held spirometer (Spirobank II^®^ Smart) and performed according to the ATS/ERS guidelines (12). The software program Winspiro was used for visualization and display of results. The results were expressed as percent predicted, according to GLI reference values (22). The best FVC and FEV_1_ of at least three technically acceptable measurements was used.

Nitrogen Multiple breath washout (MBW) measurements were performed using the Exhalyzer D (Eco Medics AG, Spiroware Version 3.2.1) and conform the consensus guidelines (13). The lung clearance index (LCI), the functional residual capacity (FRC) and the index of convection-dependent inhomogeneity multiplied by the tidal volume (s_cond_*VT) were calculated from MBW maneuvers. The mean of at least two technically acceptable measurements with maximal variation of 10% in FRC was used. Reference values for LCI provided by the software were used (Houltz and Robinson et al.). The mean normal LCI value was 6.54, the upper limit of normal 7.17. As we didn’t expect physiotherapy to have an impact on the acinar airways, we didn’t compare the change in S_acin_*VT (a measure of diffusion convention-interaction-dependent inhomogeneity in the acinar airways, distal from the terminal bronchioles, in the zone were gas exchange begins) between ACT and no ACT.

Each visit consisted of 2 MBW and 2 spirometry measurements. The lung function tests were performed in a fixed order: MBW was performed at first, followed by spirometry. Then, the intervention was performed: either a 20 min ACT session followed by a rest period of 30 min or 30 min rest without ACT. After the intervention, MBW and spirometry were repeated.

### Statistics

All variables were described using mean and standard deviation if normally distributed, otherwise median and range were used. Data were checked for normal distribution using Kolmogorov-Smirnov test. Changes in lung function parameters were calculated by substraction of the value before the intervention from the value after the intervention and reported as Δparameter. The variability of the measurements induced by the intervention was calculated as the absolute value of Δparameter, | Δparameter|. %LCI change was calculated as the relative change of LCI to control for higher variability at elevated values (23): substraction of LCI before the intervention from LCI after the intervention divided by the LCI before the intervention. Paired samples t-tests were used to assess differences within patients. Differences between both patient groups (CF and PCD) were calculated using a Mann–Whitney U test for continuous variables and Fisher’s Exact for categorical variables.

Bland-Altman plots were used to visualize the change and bias in lung function parameters induced by the intervention; the difference of the parameter before and after the intervention was plotted against the mean of the parameter before and after the intervention.

An Anova analysis with repeated measures was used to evaluate differences in lung function parameters between CF and PCD, with treatment group as within subject factor and patient group as between subject factor.

No formal power calculation was performed as we felt that no data were available in the literature to estimate the possibly expected change in lung function (both FEV_1_ and LCI) due to ACT. Therefore, the sample size was exploratory.

Statistical analysis was performed using IBM SPSS statistics version 28 and Prism version 8. A 95% confidence interval was used for all results, significance set at alfa = 0.05 without correction for multiple measures.

## Results

Thirty-three subjects (CF *n* = 19, PCD *n* = 14) were included in the study. A total of two patients with CF were excluded from the analysis (one did not show up, one received a bronchodilator during the test moment). For MBW, two additional subjects with CF were excluded from the analysis because of insufficient test repeatability. Spirometry analyses were available for 31 subjects (CF *n* = 17, PCD *n* = 14) and MBW results for 29 subjects (CF *n* = 15, PCD *n* = 14).

Baseline characteristics were similar in both groups, with a predominance of boys (Table 1). Patients with CF were significantly shorter than those with PCD. There was no difference in baseline FVC pp and FEV_1_ pp, nor in LCI between both groups.

**TABLE 1:** Baseline characteristics of the study participants.

	Total	CF	PCD	Difference (*p*-value)
Subjects (*n*)	31	17	14	
Male sex (*n*, %)	20 (65%)	11 (65%)	9 (64%)	0.636
Age (year) (mean, SD)	11.7 (3.1)	10.7 (2.8)	12.8 (3.3)	0.077
Height (z-score) (mean, SD)	−0.6 (1.0)	−1.0 (1.0)	−0.1 (0.9)	0.015
Weight (z-score) (mean, SD)	−0.6 (0.7)	−0.7 (0.7)	−0.5 (0.8)	0.653
BMI (z-score) (mean, SD)	−0.4 (0.6)	−0.2 (0.6)	−0.6 (0.6)	0.200
Bronchiectasis (*n*, %)	25 (81%)	13 (76%)	12 (86%)	0.517
FVC baseline (pp) (mean, SD)	96.6 (12.9)	96.5 (15.4)	96.6 (9.6)	0.953
FEV_1_ baseline (pp) (mean, SD)	93.0 (14.3)	94.0 (16.8)	91.9 (11.1)	0.739
LCI baseline (mean, SD)	8.6 (1.3)	8.6 (1.4)	8.6 (1.2)	0.847

There was no change in baseline characteristics between the first and the second visit: height z-score (p 0.640), weight z-score (p 0.481), BMI z-score (p 0.401), FVC pp (0.401), FEV_1_ pp (0.814), LCI (p 0.348) nor FRC (0.280) did change significantly. This was also the case when analyzing the subgroups of patients with PCD and CF separately.

**FIGURE 2:**
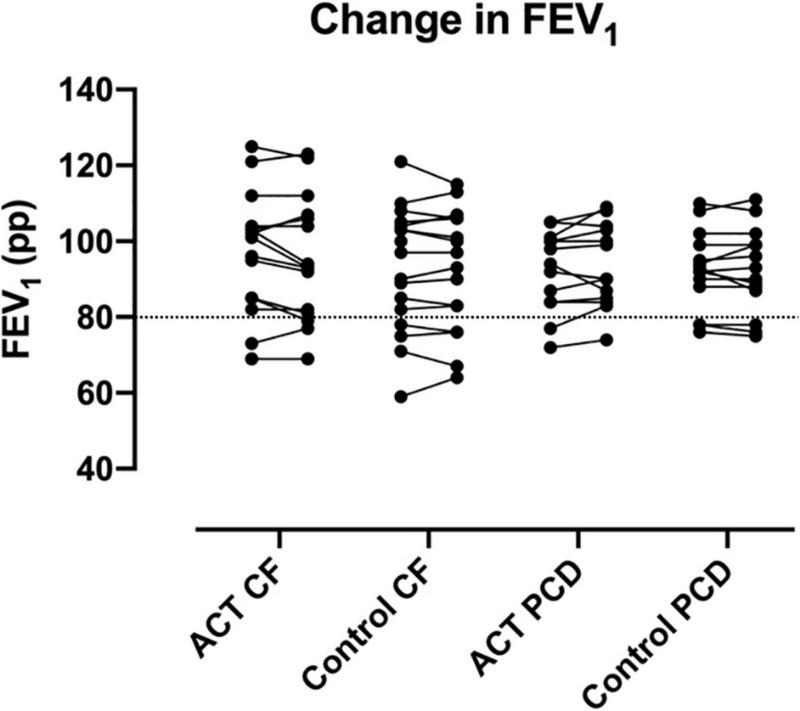
Change in FEV_1_ pp after ACT in patients with CF and PCD, compared to the control condition. No significant changes were observed, nor were significant differences observed between CF and PCD. The dashed line indicates the lower limit of normal for FEV_1_ pp.

**FIGURE 3:**
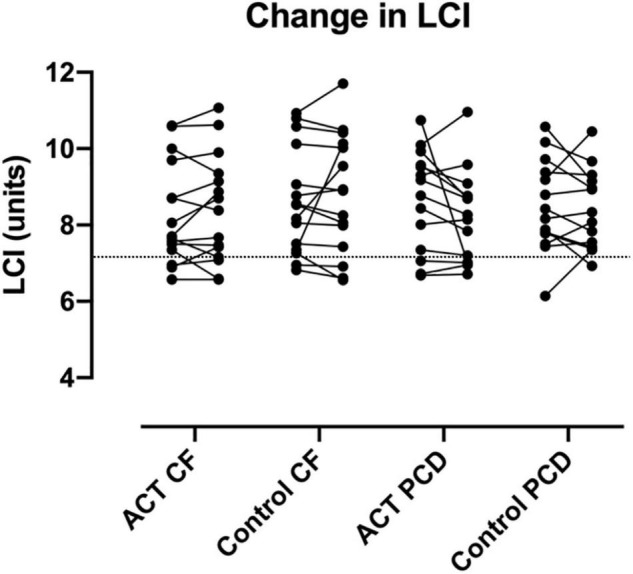
Change in LCI after ACT in patients with CF and PCD, compared to the control condition. No significant changes were observed, nor were significant differences observed between CF and PCD. The dashed line indicates the upper limit of normal for LCI.

We did not observe significant differences in ΔFVC pp, ΔFEV_1_ pp, ΔLCI, %LCI change, ΔFRC and Δs_cond_*VT between ACT and the control condition. For the total patient group (n = 31), the mean ΔFEV_1_ pp was −0.4 (range −9 to + 8) for ACT, −0.2 (range −6 to + 5) for the control condition (Figure 2); the mean ΔLCI was −0.17 (range −3.73 to + 1.17) for ACT, + 0.03 (range −1.42–+2.78) for the control condition (Figure 3). The results were similar in the subgroups of PCD and CF separately. Detailed results are presented in Table 2, graphs for ΔFVC, ΔFRC and Δs_cond_*VT are available in the online supplements. When data from patients with normal values for FEV_1_ and LCI (*n* = 5) were excluded, all comparisons remained similar (data not shown).

**TABLE 2:** Changes in lung function parameters in ACT group versus control.

	Total group (*n* = 31)			CF (*n* = 17)			PCD (*n* = 14)		
	ACT	Control	Comparison	ACT	Control	Comparison	ACT	Control	Comparison
ΔFVC pp	0.5 (3.1; −6 to + 6)	−0.7 (2.7; −6 to + 4)	0.089	0.4 (3.2; −6 to + 6)	−1.1 (3.1; −6 to + 4)	0.188	0.8 (3.2; −4 to + 6)	−0.4 (2.2; −4 to + 4)	0.313
ΔFEV1 pp	−0.4 (4.0; −9 to + 8)	−0.2 (2.8; −6 to + 5)	0.969	−1.4 (4.1; −9 to + 5)	−0.2 (3.0; −6 to + 5)	0.271	1.0 (3.7; −7 to + 8)	−0.3 (2.6; −6 to + 5)	0.293
ΔLCI	−0.17 (0.9; −3.73 to + 1.17)	0.03 (0.8; −1.42 to + 2.78)	0.294	0.10 (0.5; −0.76 to + 1.17)	0.17 (0.8; −0.47 to + 2.78)	0.814	−0.46 (1.1; −3.73 to + 0.87)	−0.11 (0.8; −1.42 to + 1.26)	0.178
%LCI change	−1.6 (8.9; −34.7 to + 15.2)	0.7 (10.5; −13.4 to + 37.8)	0.384	1.2 (6.5; −10.3 to + 15.2)	+ 2.1 (11.5; −9.6 to +37.8)	0.924	−4.6 (10.3; −34.7 to + 8.6)	−0.7 (9.4; −13.4 to + 19.7)	0.169
ΔFRC (l)	0.03 (0.2; −0.37 to + 0.74)	0.02 (0.1;−0.21 to + 0.30)	0.610	−0.10 (0.1; −0.28 to + 0.25)	0.00 (0.1; −0.08 to + 0.30)	0.841	0.08 (0.3; −0.37 to + 0.74)	0.03 (0.1; −0.21 to + 0.25)	0.405
ΔScond*VT	−0.002 (0.02; −0.06 to + 0.04)	0.003 (0.02; −0.03 to + 0.05)	0.207	0.000 (0.02; −0.03 to + 0.04)	0.002 (0.02; −0.03 to + 0.04)	0.833	−0.005 (0.02; −0.06 to + 0.04)	0.006 (0.02; −0.03 to + 0.05)	0.115

*Data are presented as mean (SD; range). Comparison between groups was performed with paired t-test.*

**FIGURE 4:**
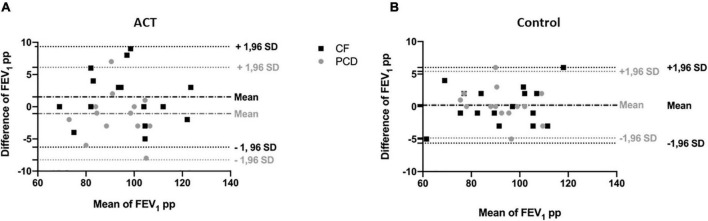
Bland and Altman plot for FEV_1_ pp in the ACT condition **(A)** compared to the control condition **(B)**, in patients with CF (black) and patients with PCD (gray). The difference of FEV_1_ pp is plotted against the mean of FEV_1_ pp to illustrate the change of the measurement after the intervention. There is no significant difference in change with ACT compared to the control condition.

**FIGURE 5:**
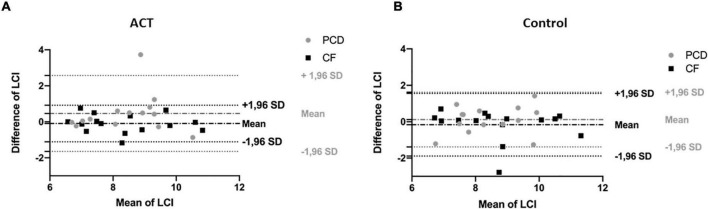
Bland and Altman plot for LCI in the ACT condition **(A)** compared to the control condition **(B)**, in patients with CF (black) and patients with PCD (gray). The difference of LCI is plotted against the mean of LCI to illustrate the change of the measurement after the intervention. There is no significant difference in change with ACT compared to the control condition.

Bland-Altman plots illustrate the change in FEV_1_ (Figure 4) and LCI (Figure 5) before and after the intervention in patients with CF and PCD, both in the ACT (a) and control group (b).

The variability of the measurements induced by the intervention were not different between the ACT and control group in the whole group of patients; |ΔFVC pp| (*p* = 0.593), |Δ FEV_1_ pp| (*p* = 0.219), |ΔLCI| (*p* = 0.721) and |Δscond*VT| (*p* = 0.106). Only |ΔFRC| was lower in the control group compared to the ACT group (*p* = 0.008). Results were similar in the subgroup of CF and PCD separately (only no significant difference in |Δ FRC| for CF).

When evaluating the difference between CF and PCD using ANOVA with repeated measures, no significant difference was found for FVC (*p* = 0.500) (effect of treatment *p* = 0.098), FEV_1_ (*p* = 0.208) (effect of treatment *p* = 0.949), neither for LCI (*p* = 0.095) (effect of treatment *p* = 0.287), FRC (*p* = 0.244) (effect of treatment *p* = 0.594) or s_cond_*VT (*p* = 0.920) (effect of treatment *p* = 0.196).

## Discussion

In this study, we observed no short-term effect of ACT on lung function parameters in children with CF and PCD. We could not confirm differences between PCD and CF.

Our findings in CF are consistent with the literature. A significant effect of ACT on FEV_1_ was found in some studies (16, 17), but only in adults with severe disease, and in combination with salbutamol administration (16). All other studies reported no effect of ACT on FEV_1_. But, only one study included a control condition to compare the variability after an intervention to the natural variability (14). A few studies also included MBW/LCI, and all showed no change after ACT. For PCD, no reliable data were available until now.

We hypothesized that ACT could potentially have a short-term effect on lung function parameters in PCD, as ACT has the aim of mechanical complementation of the impaired MCC. The hypothesis was not confirmed, possibly because all children were treated with regular ACT and evaluated outside exacerbations, and therefore only little amounts of mucus can be expectorated per session of ACT.

One of the strengths of our study is the cross-over design with the inclusion of a control condition. In this way, each patient could serve as his own control, in stable conditions. The variability induced by the ACT was compared to the variability without intervention (within-test variability) and we showed that an ACT intervention doesn’t increase the within-test variability of both FEV_1_ and LCI. Additionally, the within- and between-test variability shown in our study, was similar to what was previously shown (23). This information is important for correct interpretation of lung function parameters in long term follow-up, and for design of clinical trials where FEV_1_ and LCI are used as outcome measures.

Assignment to either ACT or control at the first visit was random. However, patients nor study personnel could be blinded for the intervention. For the first time, patients with PCD were included, and compared to patients with CF and similar disease severity. We didn’t include children with other etiologies of bronchiectasis, such as post-infectious, immune deficiency-related or idiopathic bronchiectasis. In adults with non-CF bronchiectasis (including patients with PCD), a change in LCI but not FEV_1_ after ACT has been documented (24). As non-CF non-PCD bronchiectasis may have a broad range of pathophysiological mechanism causing dysfunction of the MCC, these findings cannot be extrapolated without caution to children with non-CF non-PCD bronchiectasis.

The limitations of our study were a small study sample, and the absence of adult patients and/or patients with severe lung disease. Especially in patients with PCD and advanced disease, lung function parameters could be influenced by ACT. This should be further studied in the future. No hypertonic saline inhalation or other mucolytic was used in combination with ACT, as is often done in clinical practice for home treatment. Because we wanted to study the influence of ACT alone and because mucolytic or expectorant drugs can also influence MCC, we decided not to use inhaled drugs in combination with ACT. Because of the COVID pandemic inclusion was temporarily interrupted and several patients were not willing to participate. The time between both visits was rather long for a cross-over design, but in this way all visits could be combined with clinical visits and reduced the study burden for the patients. We realize the study was very time-consuming and tiring for the patients with at least six spirometry maneuvers and 4 MBW measurements in total per visit. Additionally, the stable baseline characteristics between both visits confirms the participating patients were stable.

Based on our findings, the timing of lung function measurements in relation to ACT doesn’t seem to have a relevant impact on the interpretation of the results in stable children. Especially for use as outcome parameter in clinical trials, no specific attention needs to be given to the timing of ACT.

## Conclusion

In this study, no significant short-term effect of ACT was found on lung function parameters in stable children with CF and PCD. Further research with a larger sample size, adult patients and other chronic lung diseases is needed.

## Data Availability Statement

The raw data supporting the conclusions of this article will be made available by the authors, without undue reservation.

## Ethics Statement

The studies involving human participants were reviewed and approved by Ethics Committee of the University Hospitals Leuven, Belgium. Written informed consent to participate in this study was provided by the participants’ legal guardian/next of kin.

## Author Contributions

BV, DD, EH, MS, and MV performed the study measurements. MB, TT, MV, and EH designed the study. MB, FV, and MP recruited the patients. BV, DD, and MB performed the statistical analysis. BV and DD drafted the manuscript. MB designed the final manuscript. All authors read and approved the final version.

## Conflict of Interest

The authors declare that the research was conducted in the absence of any commercial or financial relationships that could be construed as a potential conflict of interest.

## Publisher’s Note

All claims expressed in this article are solely those of the authors and do not necessarily represent those of their affiliated organizations, or those of the publisher, the editors and the reviewers. Any product that may be evaluated in this article, or claim that may be made by its manufacturer, is not guaranteed or endorsed by the publisher.
