# Supplementary figures and images for: The Short Term Influence of Chest Physiotherapy on Lung Function Parameters in Children With Cystic Fibrosis and Primary Ciliary Dyskinesia
Source: Front Pediatr. 2022 May 23;10:858410. doi: 10.3389/fped.2022.858410 (PMC9167999; doi:10.3389/fped.2022.858410)

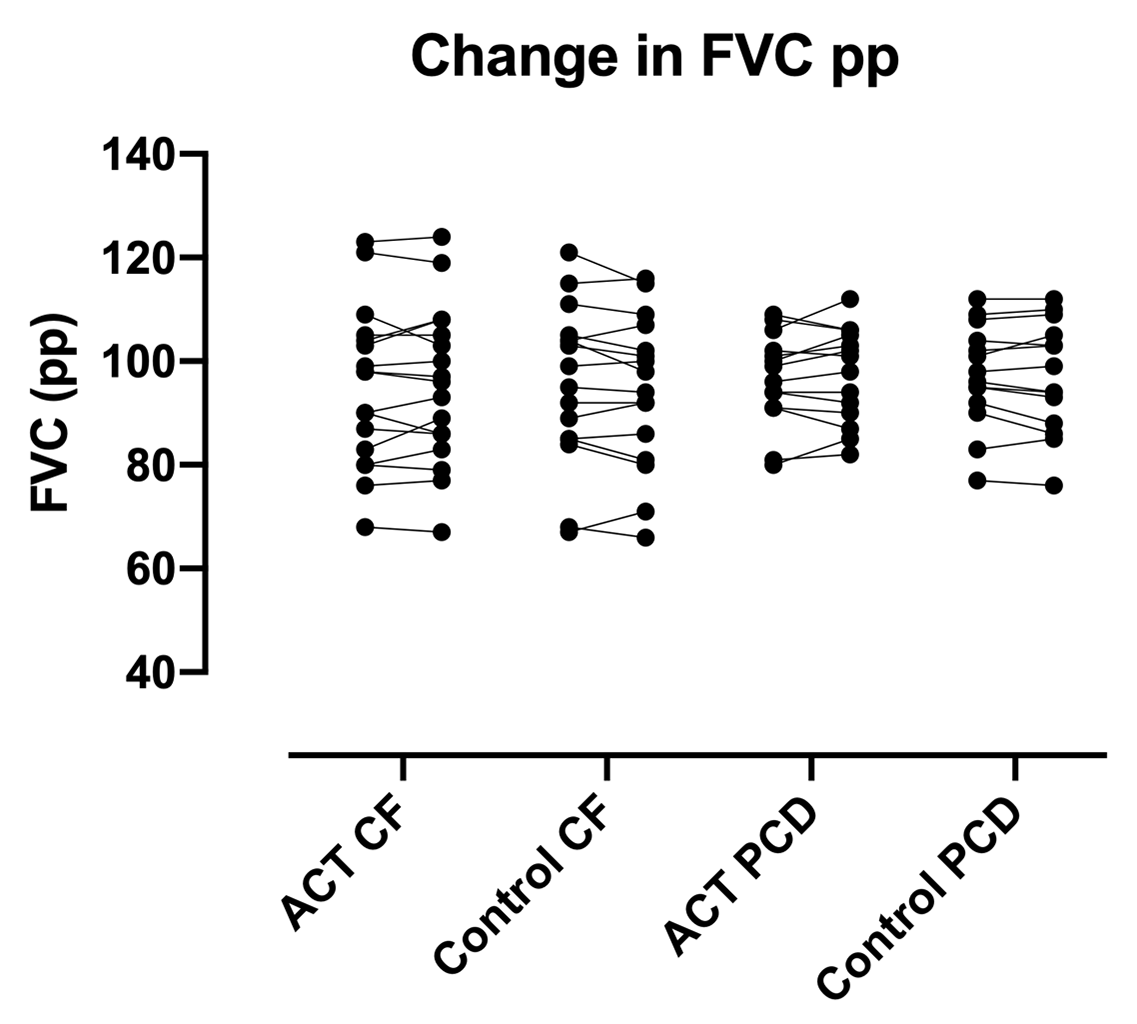

Supplement: Supplementary file 1 [file Image_1.TIFF]

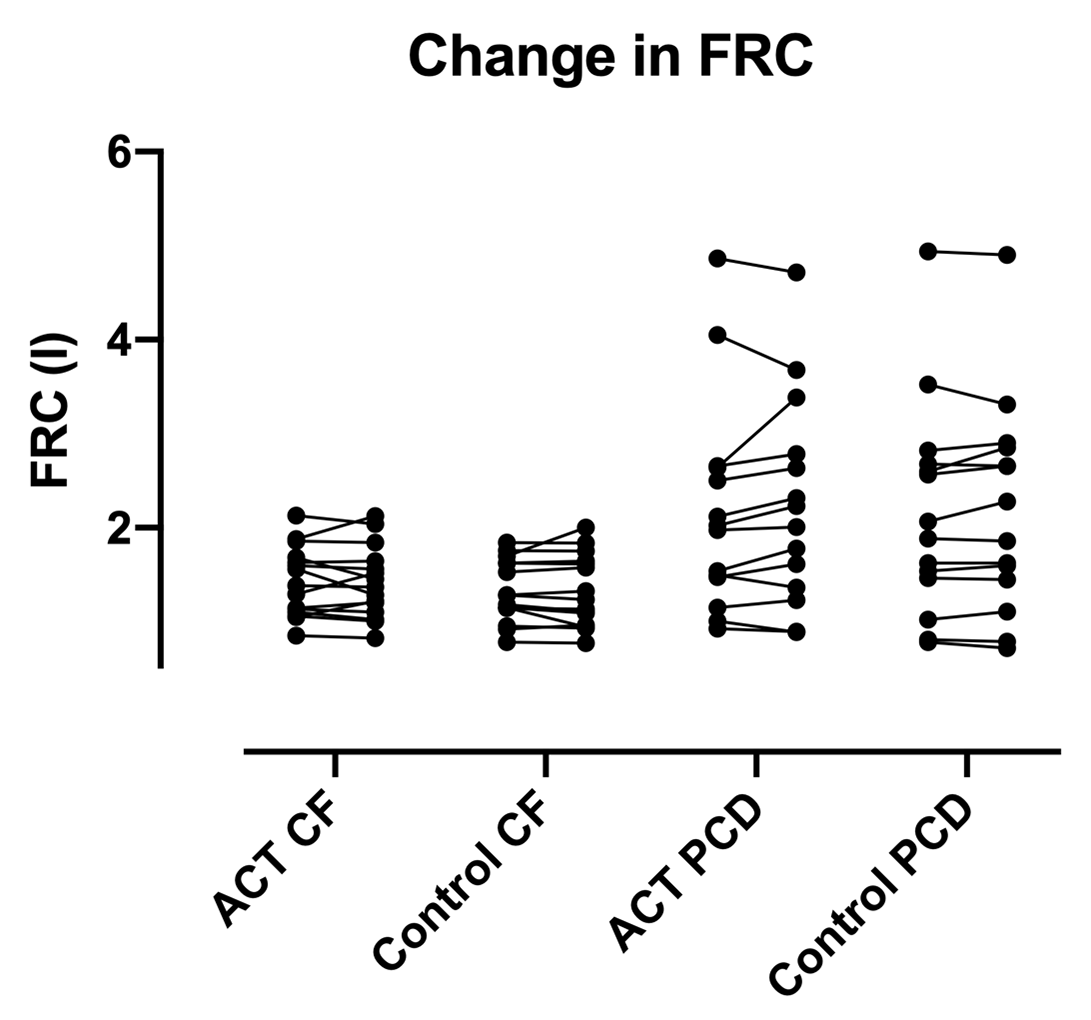

Supplement: Supplementary file 2 [file Image_2.TIFF]

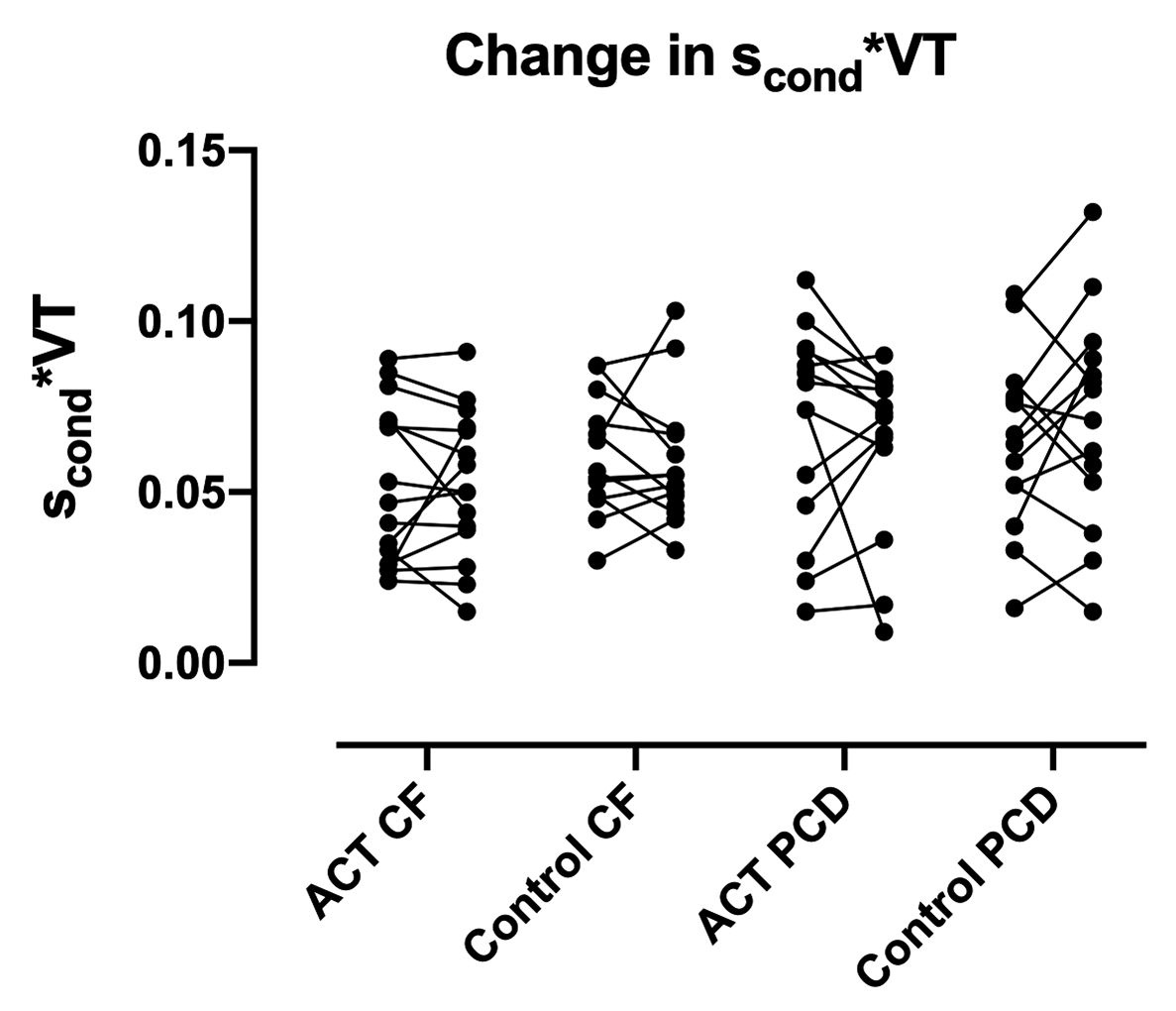

Supplement: Supplementary file 3 [file Image_3.TIFF]
