# Supplementary material for: The Short Term Influence of Chest Physiotherapy on Lung Function Parameters in Children With Cystic Fibrosis and Primary Ciliary Dyskinesia
Source: Front Pediatr. 2022 May 23;10:858410. doi: 10.3389/fped.2022.858410 (PMC9167999; doi:10.3389/fped.2022.858410)
Supplement: Supplementary file 4 [file Data_Sheet_1.docx]

Supplementary Material

# Supplementary figure 1

# Change in FVC pp after ACT in patients with CF and PCD, compared to the control condition. No significant changes were observed, nor were significant differences observed between CF and PCD.

# Supplementary figure 2

# Change in FRC after ACT in patients with CF and PCD, compared to the control condition. No significant changes were observed, nor were significant differences observed between CF and PCD.

# Supplementary figure 3

# Change in s_cond_*VT after ACT in patients with CF and PCD, compared to the control condition. No significant changes were observed, nor were significant differences observed between CF and PCD.
